# The predictive value of MRI scores for neurodevelopmental outcome in infants with neonatal encephalopathy

**DOI:** 10.1038/s41390-024-03189-1

**Published:** 2024-04-18

**Authors:** Csilla Andorka, Hajnalka Barta, Timea Sesztak, Nora Nyilas, Kata Kovacs, Ludovika Dunai, Gabor Rudas, Agnes Jermendy, Miklos Szabo, Eniko Szakmar

**Affiliations:** 1 Division of Neonatology, Pediatric Center, MTA Center of Excellence, Semmelweis University, Budapest, Hungary; 2 Department of Neuroradiology, Medical Imaging Center, Semmelweis University, Budapest, Hungary

## Abstract

**Background:**

MRI scoring systems are utilized to quantify brain injury and predict outcome in infants with neonatal encephalopathy (NE). Our aim was to evaluate the predictive accuracy of total scores, white matter (WM) and grey matter (GM) subscores of Barkovich and Weeke scoring systems for neurodevelopmental outcome at 2 years of age in infants receiving therapeutic hypothermia for NE.

**Methods:**

Data of 162 infants were analyzed in this retrospective cohort study. DeLong tests were used to compare areas under the curve of corresponding items of the two scoring systems. LASSO logistic regression was carried out to evaluate the association between MRI scores and adverse composite (death or severe disabilities), motor and cognitive outcomes (Bayley developmental index <70).

**Results:**

Weeke scores predicted each outcome measure with greater accuracy than the corresponding items of Barkovich system (DeLong tests *p* < 0.03). Total scores, GM and cerebellum involvement were associated with increased odds for adverse outcomes, in contrast to WM injury, after adjustment to 5’ Apgar score, first postnatal lactate and aEEG normalization within 48 h.

**Conclusion:**

A more detailed scoring system had better predictive value for adverse outcome. GM injury graded on both scoring systems was an independent predictor of each outcome measure.

**Impact statements:**

- A more detailed MRI scoring system had a better predictive value for motor, cognitive and composite outcomes.
- While hypoxic-ischemic brain injuries in the deep grey matter and cerebellum were predictive of adverse outcome, white matter injury including cortical involvement was not associated with any of the outcome measures at 2 years of age.
- Structured MRI evaluation based on validated scores may aid future clinical research, as well as inform parents and caregivers to optimize care beyond the neonatal period.

## Introduction

Perinatal asphyxia and consequential neonatal encephalopathy (NE) continue to be one of the leading causes of perinatal brain injury, affecting more than two million newborns worldwide.^1^ Therapeutic hypothermia (TH) decreased the incidence of adverse outcome by 19%, and thus is considered the gold standard for neuroprotection; however, approximately half of asphyxiated infants still die or develop permanent neurological impairment.^2,3^ In the last decade, several emerging neuroprotective therapies were suggested additional to TH to further reduce the risk of impairment. Nevertheless, these imply the need for early prediction of long-term outcome, to select the candidates who would benefit the most from these compound neuroprotective strategies.^4^

Magnetic resonance imaging (MRI) and proton MR spectroscopy (MRS) are among the most accurate markers to predict long-term neurodevelopmental outcome, and can successfully be used during the first weeks of life.^5,6^ Moreover, MRI can serve as a bridging biomarker and surrogate end point for long-term neurological consequences.^7,8^ To objectify the analysis of MRI images, several MRI scoring systems have been developed and validated to quantify brain injury in infants with NE, such as the Barkovich and the Weeke scoring systems.^9,10^

The Barkovich score was one of the first developed scoring systems, and initially relied on conventional MRI sequences (T1 and T2 weighted images) of the basal ganglia/thalamus (BG) and watershed areas (W).^10^ Since the introduction of diffusion weighted imaging (DWI) scans, this sequence has also been utilized in the Barkovich score. It has been extensively used for MR injury grading, especially before other scoring systems emerged, consisting of more MR modalities.^11,12^

Recently, Weeke et al. has developed a scoring system including DWI and proton magnetic resonance spectroscopy (MRS) besides conventional images, systematized into 4 subscores. Original analysis and validation of the scoring system showed the grey matter subscore (GM) to be an independent predictor of outcome at 2 years of age, while the white matter/cortex (WM) and cerebellum subscores did not add to the predictive value.^9^ There is an increasing evidence that more detailed scoring systems incorporating DWI are superior to scoring systems including conventional sequences only.^13,14^ In addition, previous studies − mostly carried out before the hypothermic era in small cohorts − had controversial results on the association between GM/BG injury and poor motor outcome, and between white matter injury and cognitive dysfunctions.^15–18^

It is crucial to clarify the association between neuroanatomic regions and neurodevelopmental domains in order to help caregivers to optimize care beyond neonatal period, as well as utilize MRI as a surrogate marker in interventional trials testing neuroprotective agents.^7,19,20^

The purpose of our study was to determine and compare the predictive accuracy of total scores, white matter and grey matter subscores of Barkovich and the Weeke scoring systems for composite, motor and cognitive outcome in infants receiving therapeutic hypothermia for NE.

## Methods

### Participants & study design

This was a single center retrospective cohort study conducted in the Division of Neonatology, Pediatric Center, Semmelweis University, Budapest, Hungary. Ethical permission for the analysis was obtained from the Scientific and Medical Research Council Ethics Committee of Hungary and consent was waived for retrospective data collection (11790-2/2016/EKU).

In our study, we reviewed neonates born between 2013 and 2019, and treated with therapeutic hypothermia for moderate-to-severe NE in our unit. All patients were outborn and admitted to our unit for cooling. Inclusion criteria were (A) gestational age ≥35 weeks, (B) provided whole body cooling as described in the TOBY (Total Body Hypothermia for Neonatal Encephalopathy) trial,^21^ (C) available post-rewarming MRI images, (D) having a neurodevelopmental follow-up examination using the Bayley Scales of Infant Development II between 18–42 months of age, as detailed below OR death. Infants with (e) postpartum asphyxia, (f) congenital malformation or (g) concurrent cerebral lesions were excluded from the study. The detailed description of clinical care including aEEG recording and scoring is described in details in Supplementary Material 1.

### Brain MRI and proton MR spectroscopy

Post-rewarming brain MRI examination was performed as part of the routine diagnostic imaging, at the Medical Imaging Center, Semmelweis University, Budapest, Hungary, using a 3 Tesla Philips Achieva scanner between 2013–2015 and 2017–2019, and on a 3 Tesla Philips Ingenia MRI scanner between 2015–2016 (Philips Medical Systems, Best, The Netherlands). Images were obtained according to a standard protocol that included MRS, T1-, T2-, DWI and susceptibility-weighted imaging (SWI), including apparent diffusion coefficient (ADC) mapping. Proton MRS were acquired using a single-voxel localization sequence (1 × 1 × 1 cm voxel in the left thalamus), at an intermediate echo time (TE) of 144 ms, repetition time (TR)  =  2000, number of signal acquisitions (NSA)  =  128.^22^

### MRI scoring

Images were scored according to Barkovich and Weeke scoring systems by two independent readers to look at interobserver variability.^9,10^ Both readers were blinded to the clinical and outcome data. The first reader (CsA) was an experienced neonatologist who was trained and supervised by a senior child neuroradiologist (GR). The second readings were carried out in the full cohort by a board-certificated radiologist specialized in neuroimaging (NNy). The interrater agreement was measured between CsA and NNy.

The Barkovich scoring system describes two patterns of brain injury: involvement of the basal ganglia, as well as involvement of watershed areas of white matter. In our study we included the basal ganglia subscore (BG), evaluating injury to the thalamus, lentiform nucleus and perirolandic cortex (max. subscore of 4), the watershed subscore (W), assessing white matter injuries in the watershed zones and cortical involvement (max. subscore of 5), as well as the summation subscore, calculated as the arithmetic sum of BG and W subscores, with maximum total score of 9. Barkovich scoring system has been utilized in neonatal encephalopathy using both conventional and diffusion weighted sequences. Consequently, DWI was also utilized when performing the Barkovich score.^11,15^

The Weeke scoring system is based on the assessment of injury to the grey matter (GM) (thalamus and basal ganglia (with additional MRS examination, accounting for 2 score points), posterior limb of the internal capsule (PLIC), brainstem, perirolandic cortex, and hippocampus; max. subscore of 25 including MRS); cerebral white matter (WM)/cortex (without perirolandic cortex, cerebral white matter, optic radiations, corpus callosum, punctate white matter lesions, and parenchymal hemorrhage; max. subscore of 21); and cerebellum (cerebellum and cerebellar hemorrhage; max. subscore of 8). The degree of injury ranges from 0 to 2 in each item. An additional subscore of 3 includes the presence of intraventricular hemorrhage (IVH), subdural hemorrhage (SDH), and sinovenous thrombosis. The total score is the sum of 4 subscores, resulting in maximum total score of 57 (in case MRS was not performed, the maximum total score is 55).^9,23^ In our study, we analyzed separately the Weeke total score with or without including the MRS abnormalities. In each scoring system, higher appointed scores indicate more extensive injury.

### Neurodevelopmental outcome

Follow-up was performed using the Bayley Scales of Infant Development II (BSID-II) toolkit, measured at 18–42 months of age by trained personnel.^24^ Composite adverse outcome was defined as either death or moderately/severely delayed development (BSID-II developmental index <70, two standard deviations below the mean score on psychomotor developmental index (PDI) or/and mental developmental index (MDI) or if assessment was not possible due to impairment severity including hearing loss requiring hearing aids or severe visual impairment). All other outcomes were considered as favorable outcome.

### Statistical analysis

Categorical variables were reported as numbers and percentages, while continuous variables as median and interquartile ranges (25^th^ to 75^th^ percentiles) or median with minimum and maximum values. Receiver-Operating Characteristics (ROC) curves were performed on all total scores as well as subscores to calculate area under the curve (AUC), optimal cut-off points and corresponding sensitivity/specificity values for predicting (a) composite adverse outcome of death and moderate to severe impairment in any domain of Bayley test, (b) cognitive (MDI < 70), as well as (c) motor (PDI < 70) impairment, separately. DeLong test was carried out to compare AUCs between subscores of corresponding brain territories: Barkovich basal ganglia and Weeke grey matter subscores (both with and without MRS), Barkovich watershed score and Weeke white matter subscores, as well as Barkovich total score (summation score) and Weeke total score (both with and without MRS). As the Barkovich system does not include assessment of cerebellar injuries, the two scoring systems were not compared from this aspect.

LASSO (Least Absolute Shrinkage and Selection Operator) regression was utilized to predict adverse composite, motor and cognitive outcome separately by selecting the subset of the variables that minimize prediction error. Variables reported in the model were automatically selected from 6 variables namely gestational age, Apgar score at 5 min, age at onset of hypothermia treatment, first postnatal pH, first postnatal lactate, aEEG normalization within 48 hours. Finally, AUCs were adjusted for the same variables selected by LASSO regression model.

Subsequently, interrater reliability was evaluated by calculation of the intraclass correlation coefficient (ICC) with a 2-way random-effects model for total scores and subscores. In addition, Bland-Altman (BA) plots were performed to assess the absolute limits of interobserver agreement between the two independent readers. In the prediction models we relied on the scores given by the first reader.

Data were analyzed using IBM SPSS Statistics software version 23.0.0.0 (IBM Corporation, Armonk, NY), as well as R Statistical software 4.0.5. (R Core Team, Vienna, Austria). The level of significance was set at 0.05.

## Results

A total of 162 newborns met all the criteria and were enrolled in this study. Patient selection is presented in Supplementary Fig. 1. Clinical characteristics of enrolled patients, as well as results of both scoring systems are presented in Table 1.

**Table 1:** Baseline characteristics and clinical data.

Variable	Study cohort *n* = 162
Gestational age (week)	39 [38; 40]
Birth weight (g)	3300 [2900; 3660]
Apgar 1 min	2 [1; 4]
Apgar 5 min	5 [3; 7]
Apgar 10 min	6 [5; 7]
First postnatal pH	7.0 [6.9; 7.2]
First postnatal base deficit (mmol/L)	17.0 [21.3; 12.5]
First postnatal lactate (mmol/L)	14 [11; 17]
Age at onset of hypothermia treatment (h)	2.1 [1.5; 3.2]
Seizures (electrical), *n* (%)	71 (44%)
Administration of phenobarbitone, *n* (%)	85 (52%)
aEEG normalization within 48 h, *n* (%)	101 (62%)
Thompson encephalopathy score^a^	11 [7; 16]
− mild encephalopathy (0–10), *n* (%)	53/119 (45%)
− moderate encephalopathy (11–14), *n* (%)	29/119 (24%)
− severe encephalopathy (15–22), *n* (%)	37/119 (31%)
MRI assessment	
Age at MRI (day of life)	4.7 [3.5; 6.2]
*Barkovich scoring system*	
Brain injury, *n* (%)	56 (35%)
Basal ganglia subscore	0 (0–4)
Watershed subscore	0 (0–5)
Summation score^b^	0 (0–9)
*Weeke scoring system*	
Brain injury, *n* (%)	115 (71%)
Grey matter without MRS subscore	0 (0–23)
Grey matter with MRS subscore^c^	0 (0–23)
White matter/cortex subscore	1 (0–17)
Cerebellum subscore	0 (0–8)
Additional score	0 (0–2)
Total score without MRS	4 (0–44)
Total score with MRS^d^	4 (0–45)
Neurodevelopmental outcomes	
Age at Bayley II test (months)	23 [19; 34]
Mental Developmental Index (MDI)	95 [84; 104]
Psychomotor Developmental Index (PDI)	95 [88; 103]
MDI < 70, *n* (%)	37/153 (24%)
PDI < 70, *n* (%)	41/153 (27%)
Death, *n* (%)	9 (5.6%)
Adverse outcome^d^	53 (33%)

Data are presented as median [IQR] or median (ranges) for MRI subscore.

*aEEG* amplitude-integrated electroencephalography, *MRS* magnetic resonance spectroscopy.

^a^Thompson score was available in 119 (73.5%) infants.

^b^Summation score is the arithmetic sum of basal ganglia and watershed subscore.

^c^MRS was performed in 138/162 (85%) of cases.

^d^Adverse outcome defined as: death OR moderately/severely delayed development (Bayley Scales of Infant Development II score <70, two SD below the mean score on PDI *or* on MDI *or* if assessment not possible due to impairment severity including hearing loss requiring hearing aids or severe visual impairment).

Brain MRI studies were carried out at a median 4.7 days of life [3.5; 6.2]. MRS was performed in 138 (85%) of cases and included in the GM subscore as well as the total score of Weeke scoring system. Presence of any brain injury (i.e. any score >0 not including the presence of SDH) was detected with higher frequency using Weeke score compared to Barkovich score (71% vs. 35%, *p* < 0.001). The median total score of Weeke with MRS was 4 (range 0–45), whereas median total score of Barkovich was 0 (range 0–9).

Interrater reliability was assessed in 157 patients to look at interobserver variability. In the remaining 5 cases discordant scores were assigned, consensus was reached in a joint reading thus these 5 MRIs were excluded from both ICC and BA analyses. The ICC for total scores and subscores of the Weeke system with the exception of cerebellum showed an excellent agreement between the two raters (Supplementary Table 2). Concerning the cerebellum subscore (max. 8 points) discordant scores (ranging between 2 and 4) were detected in 26/157 (16.6%) cases (ICC 0.557, 95% CI 0.394–0.676). There was a modest interrater agreement for the Barkovich total/summation score (ICC 0.746, 95% CI 0.650–0.815). In the Bland–Altman analysis, there were no mean differences (bias) of >1 point in either of the scores (Supplementary Table 1 and Supplementary Fig. 2). In the prediction models we relied on the scores given by the first reader.

The rate of death was 5.6% and composite adverse outcome (death or moderately/severely delayed development) occurred in 33% (53/162) of the cases. Among survivals moderately/severely delayed motor and cognitive development occurred in 27% and 24%, respectively. We need to highlight that 34/153 (22%) of infants presented with both cognitive and motor impairments (Table 1).

### White matter and grey matter injury to predict outcome

#### Composite outcome

First, we performed ROC analysis to test the diagnostic accuracy of total scores, grey matter and white matter subscores of Weeke and the identical scores of Barkovich systems to predict composite adverse outcome. In case of Weeke total score (both with and without MRS) AUC was 0.81 [95% CI 0.74; 0.88] with the optimal cutoff point of 12 (Sensitivity 57%, Specificity 93%) for adverse composite outcome, whereas based on the Barkovich total score, AUC was 0.73 [95% CI 0.66; 0.81] with the optimal cutoff point of 2 (Sensitivity 57%, Specificity 88%) (Table 2). DeLong-test showed a significant difference between the two AUCs (*p* = 0.0012) in favor of Weeke total score.

**Table 2:** Univariate analysis for diagnostic accuracy.

	Composite outcome (*n* = 53/162)				MDI < 70 (*n* = 37/153)				PDI < 70 (*n* = 41/153)			
	AUC	Cutoff	Sens.	Spec.	AUC	Cutoff	Sens.	Spec.	AUC	Cutoff	Sens.	Spec.
Weeke score												
Total score − MRS	0.81	12	57%	93%	0.80	6	73%	70%	0.79	6	68%	69%
Total score + MRS^a^	0.81	12	57%	93%	0.81	6	76%	66%	0.79	6	70%	65%
Grey matter − MRS	0.77	2	74%	72%	0.76	2	73%	66%	0.75	2	73%	67%
Grey matter + MRS^a^	0.78	1	79%	64%	0.78	1	81%	59%	0.76	2	73%	65%
White matter/cortex	0.78	3	70%	76%	0.77	3	70%	70%	0.75	3	66%	70%
Cerebellum	0.61	2	30%	92%	0.59	2	27%	88%	0.61	2	29%	89%
Barkovich score												
Total score	0.73	2	57%	88%	0.73	1	62%	74%	0.69	2	49%	81%
Basal ganglia	0.70	1	49%	89%	0.70	1	49%	84%	0.67	1	44%	83%
Watershed	0.67	2	45%	88%	0.65	2	43%	83%	0.63	2	39%	83%

Results of Receiver operating characteristics, for total scores and subscores.

*Sens.* sensitivity, *Spec.* specificity, *AUC* area under the ROC curve, *MDI* Mental Developmental Index, *PDI* Psychomotor Developmental Index, *MRS* magnetic resonance spectroscopy.

^a^MRS was performed in 138/162 (85%) of cases.

**Fig. 1:**
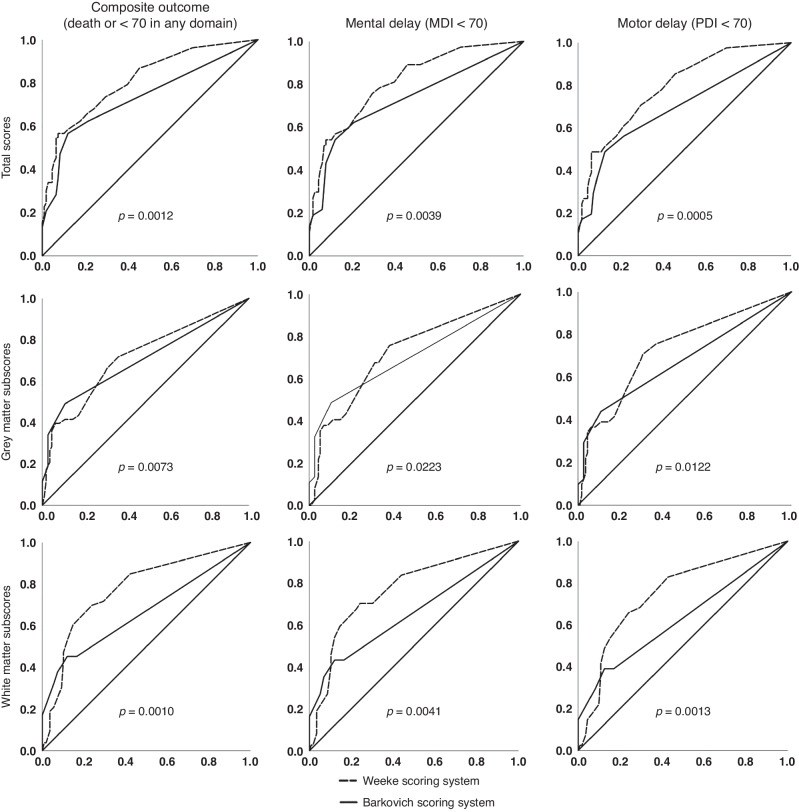
Receiver-Operating Characteristics curves demonstrating the diagnostic accuracy of corresponding subscores of Weeke and Barkovich scoring systems for adverse composite, motor and cognitive outcomes. Total score and grey matter subscore of Weeke included MRS scores as well. *P*-values are representing the results of DeLong tests that were used to compare areas under the curve of corresponding subscores. MDI mental developmental index, PDI psychomotor developmental index.

Both grey matter and white matter/cortex subscores of Weeke system provided significantly stronger prediction of composite outcome with higher AUCs, compared to Barkovich basal ganglia and watershed subscores (DeLong-tests: *p* = 0.0073 and *p* = 0.0010, respectively) (Fig. 1).

Logistic regression models were carried out to evaluate the association between MR scores and composite outcome after adjustment for clinical variables. Five minutes Apgar score, first postnatal lactate and aEEG normalization within 48 h were selected automatically from 6 covariates by LASSO regression to minimize prediction error. Both Weeke (aOR 1.10, 95% CI 1.04–1.17, *p* = 0.0027) and Barkovich total scores (aOR 1.25, 95% CI 1.03–1.55, *p* = 0.0349) were associated with increased adjusted odds ratios for composite adverse outcome.

Higher scores of grey matter injury based on both grading systems were also associated with increased adjusted odds ratios (aOR 1.16, 95% CI 1.06–1.31, *p* = 0.0048 for Weeke, and aOR 1.81, 95% CI 1.17–3.04, *p* = 0.0138 for Barkovich) for composite outcome. In contrast, white matter and watershed abnormalities including cortex involvement did not predict composite outcome in our cohort in adjusted models (Table 3).

**Table 3:** Multivariate analysis for diagnostic accuracy.

	Composite outcome (*n* = 53/162)				MDI < 70 (*n* = 37/153)				PDI < 70 (*n* = 41/153)			
	aOR	95% CI	*p* value	cAUC	aOR	95% CI	*p* value	cAUC	aOR	95% CI	*p* value	cAUC
Weeke score												
Total score − MRS	1.10	1.04 – 1.17	0.0030	0.85	1.09	1.02 – 1.17	0.0093	0.86	1.10	1.04 – 1.18	0.0029	0.82
Total score + MRS^a^	1.10	1.04 – 1.17	0.0027	0.85	1.09	1.03 – 1.16	0.0078	0.86	1.10	1.04 – 1.18	0.0026	0.81
Grey matter − MRS	1.16	1.05 – 1.31	0.0061	0.83	1.17	1.05 – 1.32	0.0082	0.84	1.19	1.07 – 1.35	0.0040	0.80
Grey matter + MRS^a^	1.16	1.06 – 1.31	0.0048	0.83	1.17	1.05 – 1.33	0.0060	0.84	1.19	1.07 – 1.35	0.0033	0.80
White matter/cortex	1.11	1.00 – 1.25	0.0555	0.84	1.10	0.99 – 1.24	0.0873	0.85	1.11	1.00 – 1.24	0.0639	0.80
Cerebellum	1.65	1.15 – 0.41	0.0072	0.85	1.60	1.07 – 2.39	0.0205	0.85	1.72	1.19 – 2.54	0.0043	0.82
Barkovich score												
Total score	1.25	1.03 – 1.55	0.0349	0.83	1.24	1.01 – 1.57	0.0487	0.84	1.23	1.01 – 1.54	0.0494	0.80
Basal ganglia	1.81	1.17 – 3.04	0.0138	0.83	1.88	1.15 – 3.32	0.0177	0.84	1.84	1.15 – 3.20	0.0172	0.80
Watershed	1.22	0.93 – 1.62	0.1589	0.83	1.23	0.92 – 1.67	0.1751	0.84	1.21	0.91 – 1.63	0.1873	0.79

Results of LASSO regression, for total scores and subscores.

*MDI* mental developmental index, *PDI* psychomotor developmental index,

*aOR* adjusted odds ratio, *CI* confidence interval, *cAUC* covariate adjusted area under the ROC-curves (covariates: Apgar 5 min., first serum lactate, amplitude-integrated electroencephalography normalization within first 48 h), *MRS* magnetic resonance spectroscopy.

^a^MRS was performed in 138/162 (85%) of cases.

ROC analysis was also adjusted for the selected covariates. Covariate-adjusted AUCs to predict composite outcome improved for each total score and subscores (Table 3).

#### Motor and cognitive outcomes

Weeke total score, grey and white matter subscores predicted motor (PDI < 70) and cognitive impairment (MDI < 70) with greater accuracy than the corresponding items of Barkovich system based on DeLong tests (Table 2 and Fig. 1).

Higher total scores in both scoring systems were associated with psychomotor and cognitive disabilities in adjusted models. Weeke grey matter scores with and without MRS results, as well as injury of basal ganglia based on Barkovich score were independent predictors of impaired motor and cognitive outcomes. White matter injuries were not associated with adverse motor, or cognitive outcomes. As for ROC analysis, the AUCs to predict MDI and PDI improved greatly for each item after covariate adjustments. See details in Table 3.

### Cerebellum

A total of 25/162 (15%) infants had cerebellar involvement, the given Weeke subscore was median 3 (range 2–8). Concomitant grey matter injuries were detected in 18/25 (72%) cases, while concomitant white matter injuries were identified in 17/25 (68%) cases. In 6 cases, the cerebellar involvement presented without concurrent deep grey matter or white matter injury. Although AUCs of cerebellum injuries were modest to predict adverse composite, motor and cognitive outcome, cerebellum subscore increased significantly the odds ratios of each outcome measurement in adjusted logistic regression models. Interestingly, the originally modest AUCs highly improved after the ROC analysis was adjusted for the selected covariates (Tables 2 and 3).

## Discussion

Based on our results, total score and each subscore of Weeke scoring system performed better in predicting adverse motor, cognitive and composite outcomes compared to the total score and corresponding subscores of Barkovich scoring system. Total scores and grey matter injury graded on each scoring system were independent predictors of each outcome measure at 2 years of age. One score increase in the total score of both systems indicated 10% rise in the adjusted OR for composite adverse outcome. In contrast, white matter abnormalities were not associated with impaired neurological outcome at this early age. Interestingly cerebellar injury was consistently predictive of motor and cognitive impairment as well as of composite outcome. However, we need to highlight that 72% of those infants presented with concomitant grey matter injury.

In agreement with our findings, Weeke et al. reported that grey matter subscore was an independent predictor of adverse composite outcome at 2 years of age; however, white matter subscore did not add to the predictive value of their regression model.^9^ Barkovich et al. showed that basal ganglia injury on conventional sequences correlated better with outcome at 3 months of age, compared to white matter injury. However, white matter score was superior to basal ganglia score to predict outcome at 12 months of age.^10^ In agreement with our findings, the study of O’Kane et al. revealed that the Barkovich basal ganglia score on both early and late MR scans (<7 and ≥7 days of age, respectively) predicted the composite outcome of motor and cognitive delay, whereas the watershed score failed to do so, irrespective of timing of MR scan, based on a small number of patients.^15^

In addition to the Barkovich and Weeke scoring systems addressed in our analysis, several other MR scoring systems have been developed. The Rutherford pattern developed for the TOBY trial was one of the first systems to grade injury severity of different brain areas, focusing on the basal ganglia/thalamus, white matter, cortex, PLIC as well as presence of intracranial hemorrhage; however, the Rutherford pattern – as indicated by its name -, did not combine its findings into a score, but instead defined the injury severity by affected cerebral territory.^25^ The score developed by the National Institute of Child Health and Human Development’s Neonatal Research Network (NICHD NRN score) includes conventional (T1- and T2-weighted) images to assess cerebral, basal ganglia/thalamic, and watershed injuries, combining them into one final score of 6 severity grades, so has no separate assessment of grey and white matter lesions.^26^ The Trivedi score on the other hand, analyses T1-, T2-weighted and DWI images, as well, and grades abnormalities of the deep nuclei+PLIC, white matter, cortex, cerebellum and brainstem separately, resulting in 5 individual subscores, that can be further summed up to get the cumulated score.^27^

Despite the large number of available grading scales, we decided to compare the accuracy of the most recent and detailed scoring system developed by Weeke et al., and the widely used MR scoring system describing the two classic patterns of hypoxic- ischemic brain injury (deep grey matter predominant as well as watershed), published by Barkovich et al.

Some studies have previously attempted to assess the predictive accuracy of various MR scoring systems. Preeminently, we need to point out that the definition of adverse outcome varies between each article. From these, Langeslag et al. conducted the largest study so far, analyzing 161 newborns, whose brain MR images have been scored using the Trivedi, NICHD NRN, Weeke and Rutherford total scores. The analysis revealed that Weeke total score demonstrated an AUC of 0.75 for the prediction of adverse motor and/or cognitive outcome and an AUC of 0.91 for composite outcome of death and adverse outcome after adjusting the model for birthweight, Thompson encephalopathy score, seizures confirmed by aEEG and baseline pH.^28^ Bhroin et al. included 66 infants with MRI and follow-up data in the final analysis aiming to assess the relationship between three MRI scoring systems and Bayley-III score of cognitive, motor and language outcome separately. This study also confirmed the association between total score of Weeke and combined assessment of basal ganglia and watershed injury by Barkovich and motor, cognitive outcomes, providing the R^2^ values of multiple linear regression. In concordance with our findings, the Weeke scoring system detected brain injury with the highest frequency compared to other scoring systems.^14^ Furthermore, Weeke scoring system was sensitive to identify atypical brain injuries in patients with mild hypoxia-ischemia.^13^ The reason behind this phenomenon can be that Barkovich et al. described two typical patterns of brain abnormalities that were linked to acute profound and partial prolonged asphyxia whereas Weeke et al. outlined a more extended scoring system, which was capable to detect atypical forms of hypoxic-ischemic brain injury and additional MRI findings.^6^ The differences described above between the two scoring systems could explain the higher aOR of Barkovich compared to Weeke scores in the multiple logistic regression models. Barkovich score describes the typical and more severe brain abnormalities resulting in a maximum total/summation score of 9, whereas the Weeke score can detect a broad pattern of brain injury, with a maximum total score of 57.

In addition to the above cited studies, we aimed to evaluate the predictive value of different patterns of brain injury (white and grey matter, cerebellum) for composite outcome (death or adverse motor/cognitive outcome), as well as for motor and for cognitive delays, separately. The association between pattern of injury and domains of impairment has been widely studied, with controversial results. In some previous studies, abnormal signal intensity in grey matter was associated with poor neuromotor outcomes,^29,30^ whereas watershed injury was linked to impaired language skills, behavioral problems and epilepsy in later childhood.^16–18,31^ The small prospective study of Miller et al. demonstrated that the watershed pattern of injury was associated with cognitive deficits at 30 months, while cognitive delays were underestimated at 12 months of age.^16^ Steinman et al. revealed an independent association between lower verbal IQ and watershed injury, while neither watershed nor BG injuries were predictive of non-verbal cognitive abilities such as visuospatial knowledge, visual memory, and executive function at 4-years of age, before the hypothermic era.^17^ Data from the same research group demonstrated an association between watershed predominant pattern of injury on neonatal MRIs and lower overall cognitive ability, Perceptual Reasoning Index, and digit span score at age of 10–16 years in 7 patients. They concluded that worse language outcome in early childhood may be linked to decreasing cognitive ability in adolescence.^32^

Our results did not confirm the association between white matter injury and cognitive outcome, whereas grey matter involvement was significantly predictive for both domains. Given our criteria for adverse outcome (developmental index < 70), it is considered unlikely that severe or moderate delay in cognitive domain was overlooked at 2 years of age.

The lack of association between white matter injuries and neurodevelopmental outcome can be explained by the broad age range of performing neurodevelopmental examination [IQR 19; 34] due to difficulties with patient compliance, and strict cutoff values on Bayley- II test, which we defined.

Furthermore, the absence of association between signal abnormalities in specific brain regions and type of impairments can be due to the high co-occurrence of motor and cognitive delays as well as grey matter and white matter injuries in our cohort. In addition, we need to point out that language skills were not assessed separately in our cohort.

Although the cerebellum is not considered among the classical brain territories susceptible to hypoxia-ischemia, growing evidence suggest its involvement in NE is severely underestimated.^33^ A study examining 33 infants with moderate-to-severe NE revealed decreased ADC values in the cerebellum (especially the cerebellar vermis and dentate nuclei), which was correlated to the number of normal Purkinje cells visible on histopathological findings, compared to non-NE controls, even though no abnormality was visible on DWI scans.^34^ Another research based on 57 asphyxiated newborns discovered that while qualitative analysis of conventional and diffusion tensor images (DTI) showed no evidence of cerebellar involvement, quantitative scalars such as fractional anisotropy (FA) and mean diffusion (MD) were significantly lower in cerebellar peduncles.^33^ This finding is corroborated by Kwan et al., who analyzed 172 infants with moderate-to-severe NE treated with hypothermia. Based on their results, while only 4% of patients showed cerebellar injury on conventional MR images, quantitative measures such as ADC and FA were significantly lower in cerebellar peduncles and dentate nuclei in the presence of visible cerebral injury, and even lower in cases where cerebellar injury was detected on MR images.^35^ This implies that cerebellar injury is often overlooked on conventional MRI scans, and can mostly be detected using quantitative measures of diffusion (ADC, FA and MD scalars). Consequently, the prevalence of cerebellar involvement in NE could be highly underestimated.

Our results seem to comply with and be explained by the above-mentioned findings. Even though AUCs of Weeke cerebellum subscore were modest to predict adverse outcome, they highly improved after adjustment to selected clinical variables. Moreover, in the adjusted logistic regression models, cerebellum subscore augmented the odds ratio of each outcome.

Previously, it has been hypothesized that cerebellar injury is usually a manifestation of extremely severe deep grey matter involvement.^36,37^ The high percentage (72%) of concomitant grey matter and cerebellar injury in our cohort could explain the strong effect of cerebellum involvement on neurological outcome. We speculate that the association between increasing cerebellum subscore and adverse outcome may be caused, at least in part, by underlying grey matter injury. Clearly, further investigations of this injury pattern are needed, especially considering how often cerebellar involvement is overlooked on conventional MRI sequences, as we have detailed above.

The interobserver reliability between the 2 readers was consistent with previously reported data.^38^

Limitations of our study include the retrospective study design, carrying the inherent bias of having to rely on previously recorded patient documentation. Second, cortical involvement was incorporated in white matter injury scores indicated by the evaluated scoring systems. Thus, we did not analyze cortex and white matter abnormalities separately. Also, we decided to use the summation score of Barkovich system to be comparable with Weeke total score, even though it was previously established that the combination of basal ganglia/watershed abnormalities had a better predictive value for long term outcomes, compared to the arithmetical sum of subscores.^10^ Third, due to resource availability, two different 3 Tesla MRI scanners were used to examine our patient population; however, as magnetic field strength and acquisition protocols were identical, this should not bias significantly the scoring of MR images. Lastly, we need to highlight that patterns of brain injury as well as neurological dysfunctions in motor and cognitive domains overlapped in high percentage. Addressing the question of link between specific neuroanatomic regions of the brain and domain-specific outcome measures would require a larger sample size.

So far, this cohort is one of the largest to compare the diagnostic accuracy of two widely accepted MRI scoring systems and to investigate the associations between different patterns of brain injuries and neurodevelopmental outcomes. To ensure the accuracy of observed relations, multiple statistical methods were applied.

## Conclusion

A more detailed scoring system incorporating DWI and proton MR spectroscopy has a better predictive value for adverse outcome in infants with NE. Our results confirmed the previous findings that grey matter abnormalities are highly predictive of neurological outcomes at 2 years of age. However, the lack of association between white matter/cortex injuries and adverse outcome at 2 years needs further evaluation, and so does cerebellar involvement and its effect on long-term prognosis in this vulnerable patient population.

Overall, MRI can not only serve as a bridging biomarker and a surrogate end point for neurodevelopmental outcome, but also inform parents and caregivers, and help optimize care beyond the neonatal period. Consequently, quantifying brain injuries based on standardized scoring systems is crucial.

## Supplementary information

Supplementary Figure 1

Supplementary Figure 2

Supplementary Material 1

Supplementary Tables 1, 2

## Data Availability

The datasets generated during and/or analyzed during the current study are available from the corresponding author on reasonable request.
