# Supplementary material for: The predictive value of MRI scores for neurodevelopmental outcome in infants with neonatal encephalopathy
Source: Pediatr Res. 2024 Apr 18;97(1):253–60. doi: 10.1038/s41390-024-03189-1 (PMC11798823; doi:10.1038/s41390-024-03189-1)
Supplement: Supplementary file 1 — Supplementary Figure 1 [file 41390_2024_3189_MOESM1_ESM.pdf]

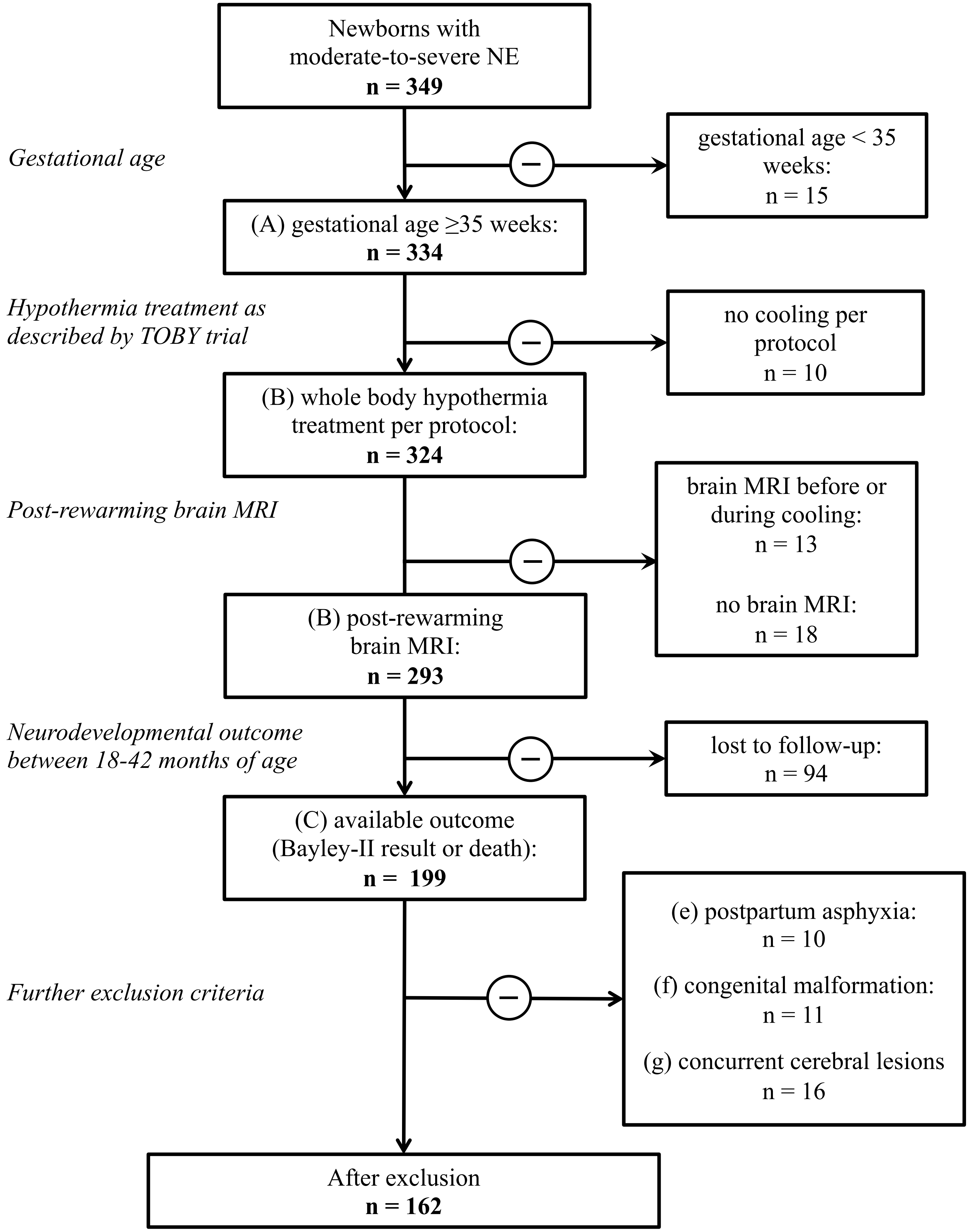

Newborns with moderate-to-severe NE
n = 349
Gestational age
gestational age < 35 weeks:
n = 15
(A) gestational age ≥35 weeks:
n = 334
Hypothermia treatment as described by TOBY trial
no cooling per protocol
n = 10
(B) whole body hypothermia treatment per protocol:
n = 324
Post-rewarming brain MRI
brain MRI before or during cooling:
n = 13
no brain MRI:
n = 18
(B) post-rewarming brain MRI:
n = 293
Neurodevelopmental outcome between 18-42 months of age
lost to follow-up:
n = 94
(C) available outcome (Bayley-II result or death):
n = 199
Further exclusion criteria
(e) postpartum asphyxia:
n = 10
(f) congenital malformation:
n = 11
(g) concurrent cerebral lesions
n = 16
After exclusion
n = 162
