# Supplementary material for: The predictive value of MRI scores for neurodevelopmental outcome in infants with neonatal encephalopathy
Source: Pediatr Res. 2024 Apr 18;97(1):253–60. doi: 10.1038/s41390-024-03189-1 (PMC11798823; doi:10.1038/s41390-024-03189-1)
Supplement: Supplementary file 2 — Supplementary Figure 2 [file 41390_2024_3189_MOESM2_ESM.pdf]

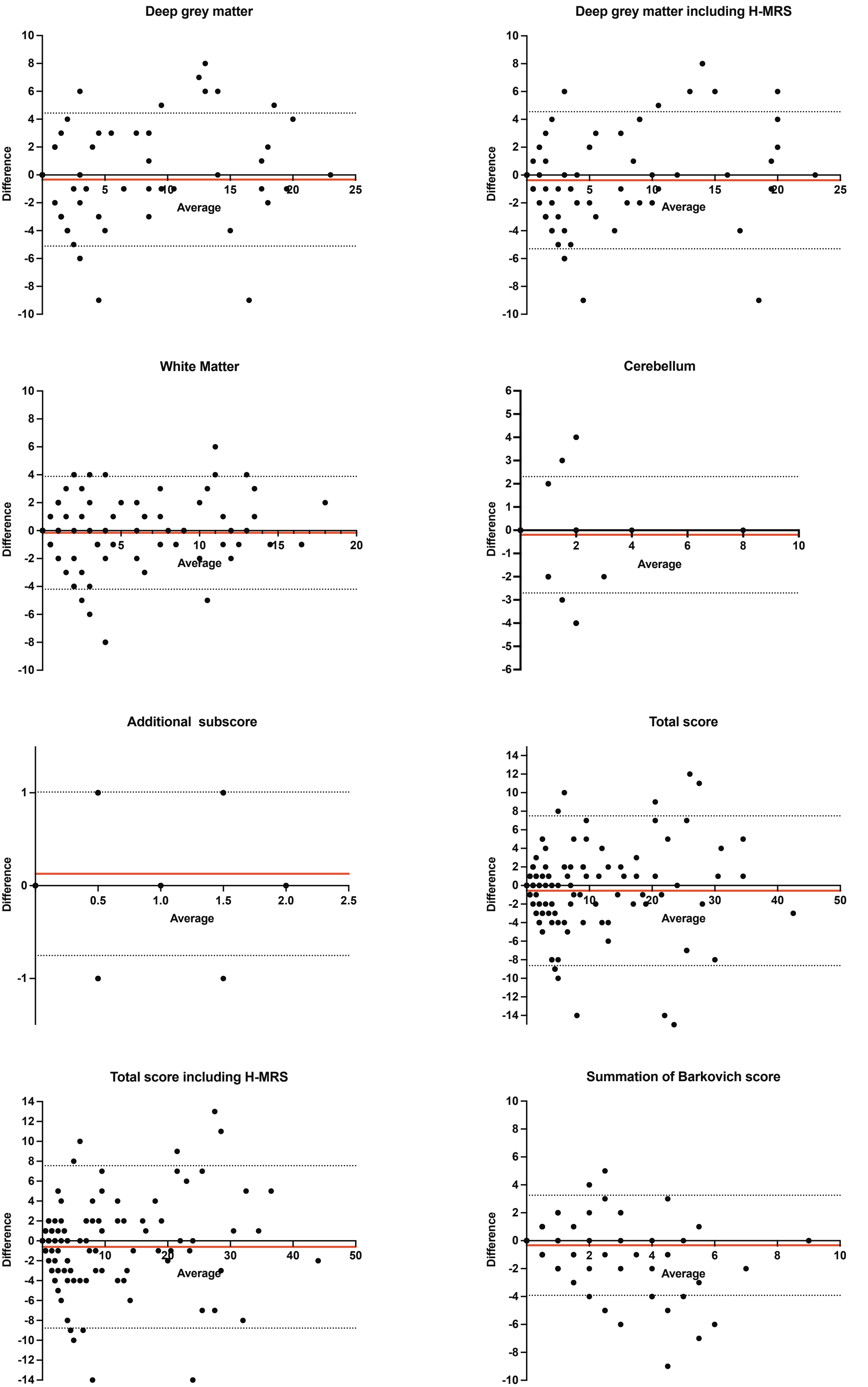
Deep grey matter
Deep grey matter including H-MRS
White Matter
Cerebellum
Additional subscore
Total score
Total score including H-MRS
Summation of Barkovich score
Difference
Average
