# Supplementary material for: The predictive value of MRI scores for neurodevelopmental outcome in infants with neonatal encephalopathy
Source: Pediatr Res. 2024 Apr 18;97(1):253–60. doi: 10.1038/s41390-024-03189-1 (PMC11798823; doi:10.1038/s41390-024-03189-1)
Supplement: Supplementary file 3 — Supplementary Material 1 [file 41390_2024_3189_MOESM3_ESM.pdf]

Supplementary Material 1.

Clinical care of infants receiving therapeutic hypothermia

Whole-body cooling was induced as soon as possible, but within the first 6 hours of life, using water-filled mattress (Tecotherm; TecCom, Halle, Germany) maintained for 72 hours, followed by gradual rewarming. The target rectal temperature was between 33 and 34 °C. During rewarming, the temperature increase velocity was 0.5 °C/h. All patients were mechanically ventilated throughout the hypothermic and rewarming phase. Sedation was provided using continuous infusion of morphine, and clinical or electrophysiological seizures were treated with phenobarbitone, midazolam or levetiracetam, if necessary.

Cerebral activity was recorded using continuous single-channel (biparietal, P3-P4) aEEG monitoring (Olympic CFM 6000 monitor; Natus Medical or EEG-1200K; Nihon Kohden). Recording was started as soon as possible but always before 6 hours of age and continued for the duration of therapeutic hypothermia and rewarming, for a total of 84 hours of study [1]. The aEEG background activity was scored for each 6-hour interval by 2 neonatologists using the Hellström–Westas criteria [2]. The first appearance of sustained CNV or DNV (lasting over a 3-hour period) on the aEEG was defined as aEEG recovery [1]. Neurological staging was performed using the Thompson score [3].

References:

1 Meder, U. et al. Longitudinal Analysis of Amplitude-Integrated Electroencephalography for Outcome Prediction in Hypoxic-Ischemic Encephalopathy. *J Pediatr* **246**, 19-25 e15 (2022).

2 Hellstrom-Westas, L., Rosen, I. & Svenningsen, N. W. Predictive Value of Early Continuous Amplitude Integrated Eeg Recordings on Outcome after Severe Birth Asphyxia in Full Term Infants. *Arch Dis Child Fetal Neonatal Ed* **72**, F34-38 (1995).

3 Thompson, C. M. et al. The Value of a Scoring System for Hypoxic Ischaemic Encephalopathy in Predicting Neurodevelopmental Outcome. *Acta Paediatr* **86**, 757-761 (1997).

Supplementary figure 1. Patient selection algorithm.

NE: neonatal encephalopathy, TOBY: Total Body Hypothermia for Neonatal Encephalopathy, MRI: magnetic resonance imaging

Supplementary Figure 2. Bland-Altman plots for total scores and subscores.

The red line indicates bias (mean difference), whereas dashed lines shows 95% limits of agreement.
