# Supplementary material for: The predictive value of MRI scores for neurodevelopmental outcome in infants with neonatal encephalopathy
Source: Pediatr Res. 2024 Apr 18;97(1):253–60. doi: 10.1038/s41390-024-03189-1 (PMC11798823; doi:10.1038/s41390-024-03189-1)
Supplement: Supplementary file 4 — Supplementary Tables 1, 2 [file 41390_2024_3189_MOESM4_ESM.pdf]

Supplementary Table 1. Results of Bland-Altman analysis representing the absolute limits of interobserver agreement between the 2 readers.

	Weeke scoring system							Barkovich
	Deep grey matter	Deep grey matter with H-MRS	White matter	Cerebellum	Additional	Total score without H-MRS	Total score including H-MRS	Summation
Bias	-0.3376	-0.3758	-0.1592	-0.1975	0.1274	-0.5669	-0.6115	-0.3312
SD of bias	2.435	2.513	2.062	1.278	0.4490	4.114	4.166	1.830
95% limits of agreement								
From	-5.111	-5.300	-4.200	-2.703	-0.7527	-8.631	-8.777	-3.917
To	4.435	4.549	3.881	2.308	1.007	7.497	7.554	3.255

H-MRS, proton magnetic resonance spectroscopy; SD standard deviation
Bland-Altman analysis was performed in 157/162 (96.9%) of cases to assess the absolute limits of interobserver agreement; in the remaining 5 cases a consensus reading was carried out and these cases were excluded from the present analysis. Bland-Altman plots are presented in supplementary figure 2.

Supplementary Table 2. Interrater reliability between the 2 readers.

	ICC	95% Confidence interval
Deep grey matter	0.944	0.923–0.959
Deep grey matter with H-MRS	0.947	0.928–0.962
White matter	0.943	0.922–0.958
Cerebellum	0.557	0.394–0.676
Additional	0.833	0.762–0.882
Total score without H-MRS	0.947	0.928–0.962
Total score with H-MRS	0.950	0.931–0.964
Summation (Barkovich score)	0.746	0.650–0.815

H-MRS, proton magnetic resonance spectroscopy; ICC, intraclass correlation coefficient
Interrater reliability was evaluated by calculation of the ICC with a 2-way random-effects model. ICC was calculated in 157/162 (96.9%) of cases; in the remaining 5 cases a consensus reading was carried out and these cases were excluded from the present analysis.
